# Supplementary material for: Caloric Restriction Reprograms Adipose Tissues in Rhesus Monkeys
Source: Aging Cell. 2025 Oct 3;24(12):e70254. doi: 10.1111/acel.70254 (PMC12686577; doi:10.1111/acel.70254)
Supplement: Supplementary file 1 — Appendix S1: acel70254‐sup‐0001‐Supinfo1.pdf. [file ACEL-24-e70254-s004.pdf]

AGING CELL AUTHOR CHECKLIST. ***Authors should submit this checklist together with their manuscript. Please ensure that you have read the Author Guidelines in detail before submission.***

**Title**	Caloric restriction reprograms adipose tissues in rhesus monkeys
**Authors**	Josef P Clark, Timothy W Rhoads, Sean J McIlwain, Michael A Polewski, Derek M Pavelec, Ricki J Colman, Rozalyn M Anderson
**Manuscript Type**	Research Article
**Total Character Count (including spaces)[1]**	45,620
**Word count of Summary[2]**	146
**Number of papers cited in the References[3]**	94
**Listing of all Tables (Table1, Table 2 etc)[4]**	N/A

**Figure specifications (please complete one row per figure)[5]** ***Figure no.***	Colour ***(yes/no)***	Greyscale ***(yes/no)***	Black and white ***(yes/no)***	Single column (80mm) ***(yes/no)***	Double column (180mm) ***(yes/no)***	Size of figure at full scale (mm x mm) ***(insert details)***	Smallest font size used in the figure at full scale (minimum 6pt) ***(insert details)***
Figure 1	yes	no	no	no	yes	192 x 206	6pt
Figure 2	yes	no	no	no	yes	191 x 148	6pt
Figure 3	yes	no	no	no	yes	190 x 163	6pt
Figure 4	yes	no	no	no	yes	172 x 255	6pt
Figure 5	yes	no	no	yes	no	122 x 254	6pt

[1] The maximum character count allowed is 50,000 (incl. spaces) for Primary Research Papers and Reviews, 10,000 for Short Takes.
[2] Summary should not exceed 250 words.
[3] Primary Research Papers can contain a maximum of two tables. If more are needed they should replace some of the Figures or can be placed in the Supporting Information.
[4] A maximum of 45 references is allowed for Primary Research Papers and 20 references for Short Takes.
[5] A Primary Research Paper may contain up to 6 figures and a Short Take up to 2 figures. Authors are encouraged to provide figures in the size they are to appear in the journal and at the specifications given.
