# Supplementary material for: Caloric Restriction Reprograms Adipose Tissues in Rhesus Monkeys
Source: Aging Cell. 2025 Oct 3;24(12):e70254. doi: 10.1111/acel.70254 (PMC12686577; doi:10.1111/acel.70254)
Supplement: Supplementary file 4 — Figure S1: Blood analyses and biometric data for individuals on study. (A) Graphs showing biometric and blood chemistry measures for individuals on the Control or CR diet (n = 4; bar, mean; line, SD). Significance indicated by * (p < 0.05) or p‐value listed from student t‐test. Measures include: age, weight, fat, lean, appendicular lean mass, total and total abdominal fat percent, HOMA‐IR, glycosylated‐hemoglobin (Glyc HGB), basal glucose and insulin, insulin sensitivity (Si), cholesterol, triglycerides, white and red blood cell counts (WBC and RBC), hemoglobin (Hgb), hematocrit (Hct), blood urea nitrogen (BUN), creatinine, lactate dehydrogenase (LDH), gamma‐glutamyl transferase (GGT), alanine aminotransferase (ALT), aspartate aminotransferase (AST), alkaline phosphatase (ALP), total protein, albumin, total bilirubin. Figure S2: Depot‐specific expression of Adipokines and SenNet factors. Heatmaps of SAT/VAT Log2FC for Adipokines (left) and SenNet factors (right). Dot denotes significance. Related to Figure 1. Figure S3: Depot‐specific transcriptional response to CR. (A) Bar chart depicting the Log2FC (CR/C) of DEGs that are shared or unique to the three comparisons: both depots (gray), SAT (yellow) or VAT (blue). (B) Principal component analysis plot of the transcriptome for each group (n = 4). Ellipse denotes 80% confidence level. Related to Figure 2. Figure S4: WGCNA module‐trait associations. Correlation of module eigengenes with individual biometric measures (traits). Boxes contain module‐trait correlations (top) and p‐value (bottom). Numbers of genes per module are listed on the right. Related to Figure 4. Figure S5: Blue module trait associations enrich for oxidative phosphorylation gene containing pathways. (A) Scatter plots of gene significance versus blue module‐membership for genes associated with triglycerides, lean and appendicular mass, and insulin sensitivity (Si). n (number of genes), cor (correlation), p (pvalue); (B) Bar plot of KEGG pathways enrich [file ACEL-24-e70254-s001.pdf]

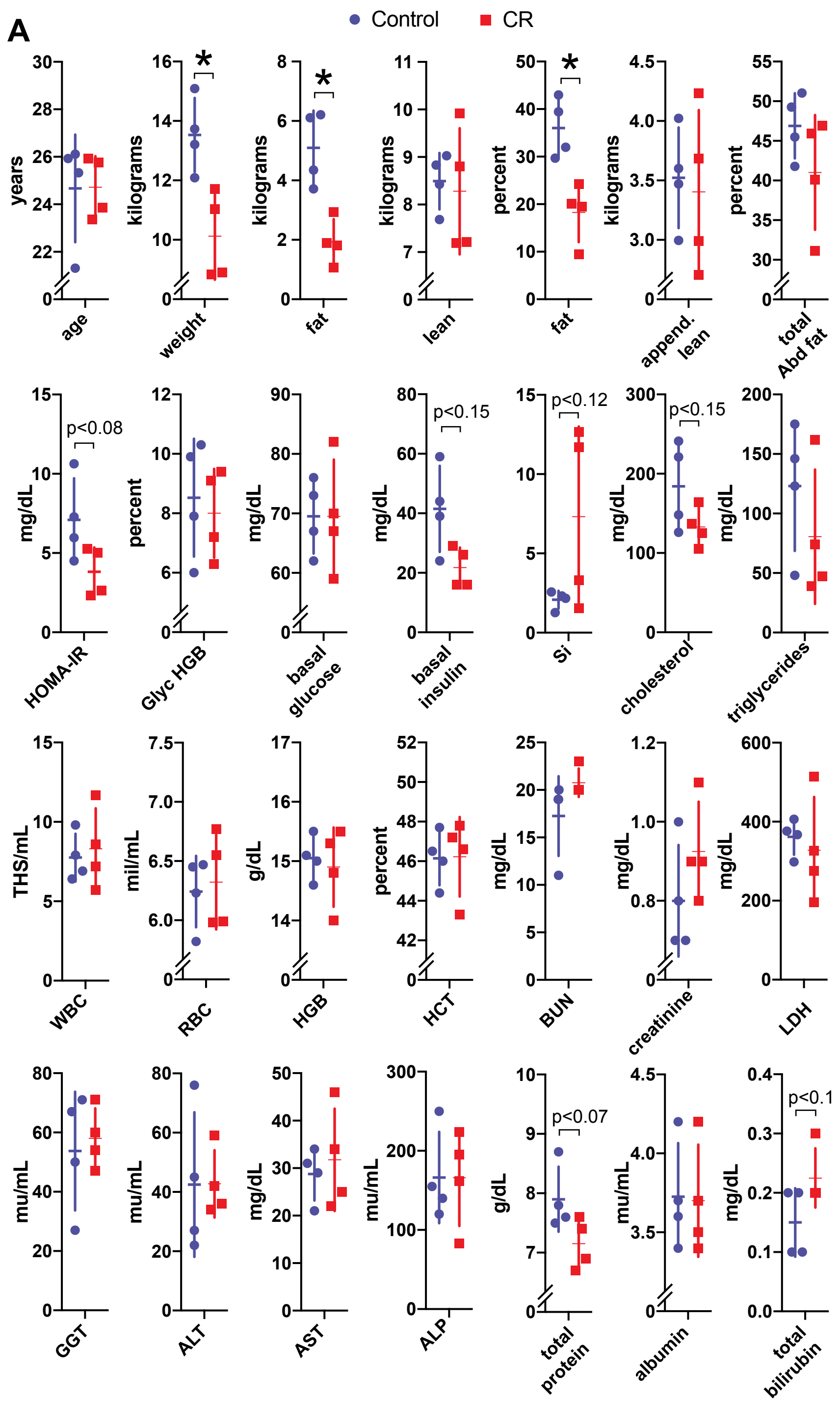

Supplementary Figure S1

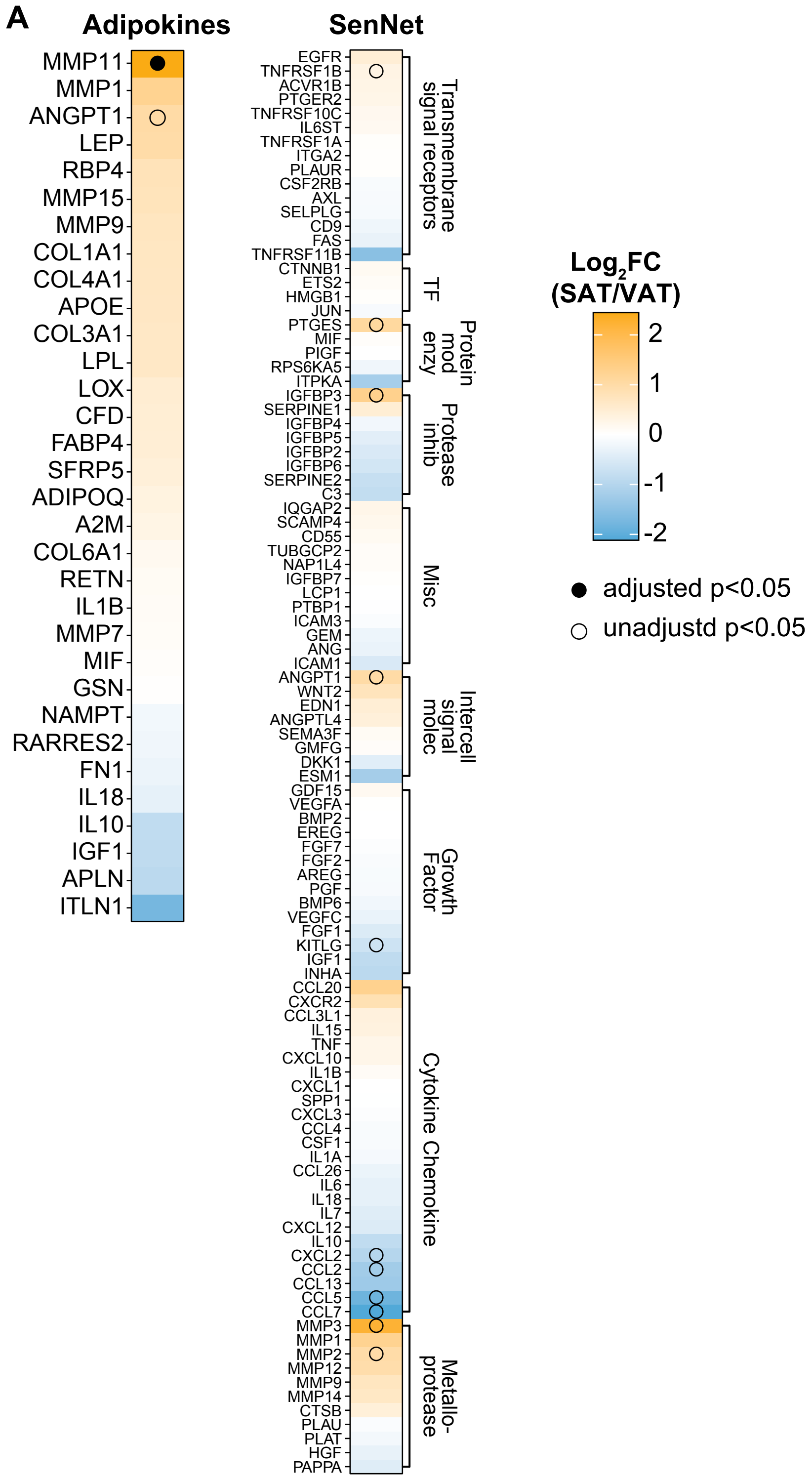

Supplementary Figure S2 (Related to Figure 1)

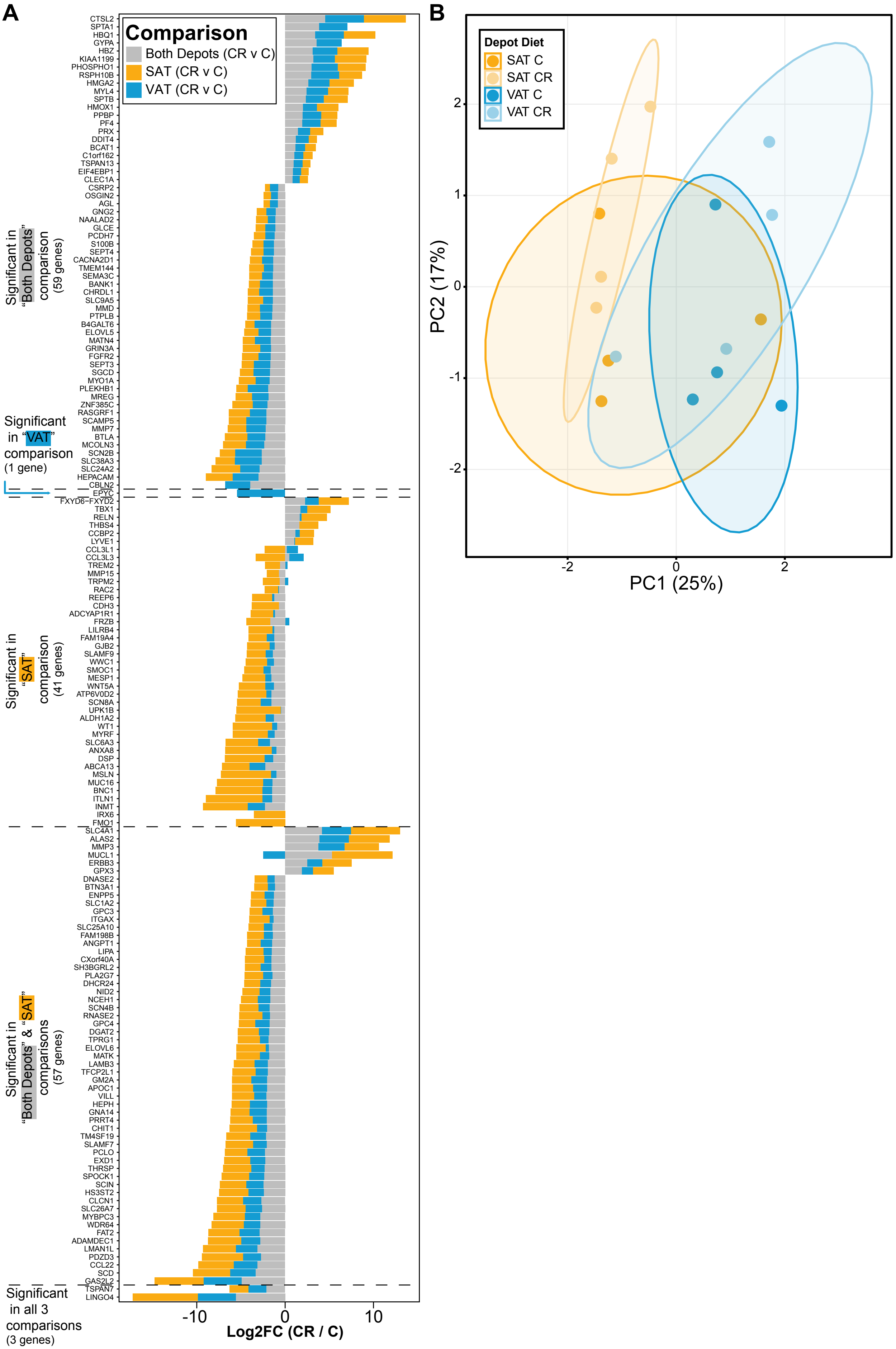

Supplementary Figure S3 (Related to Figure 2)

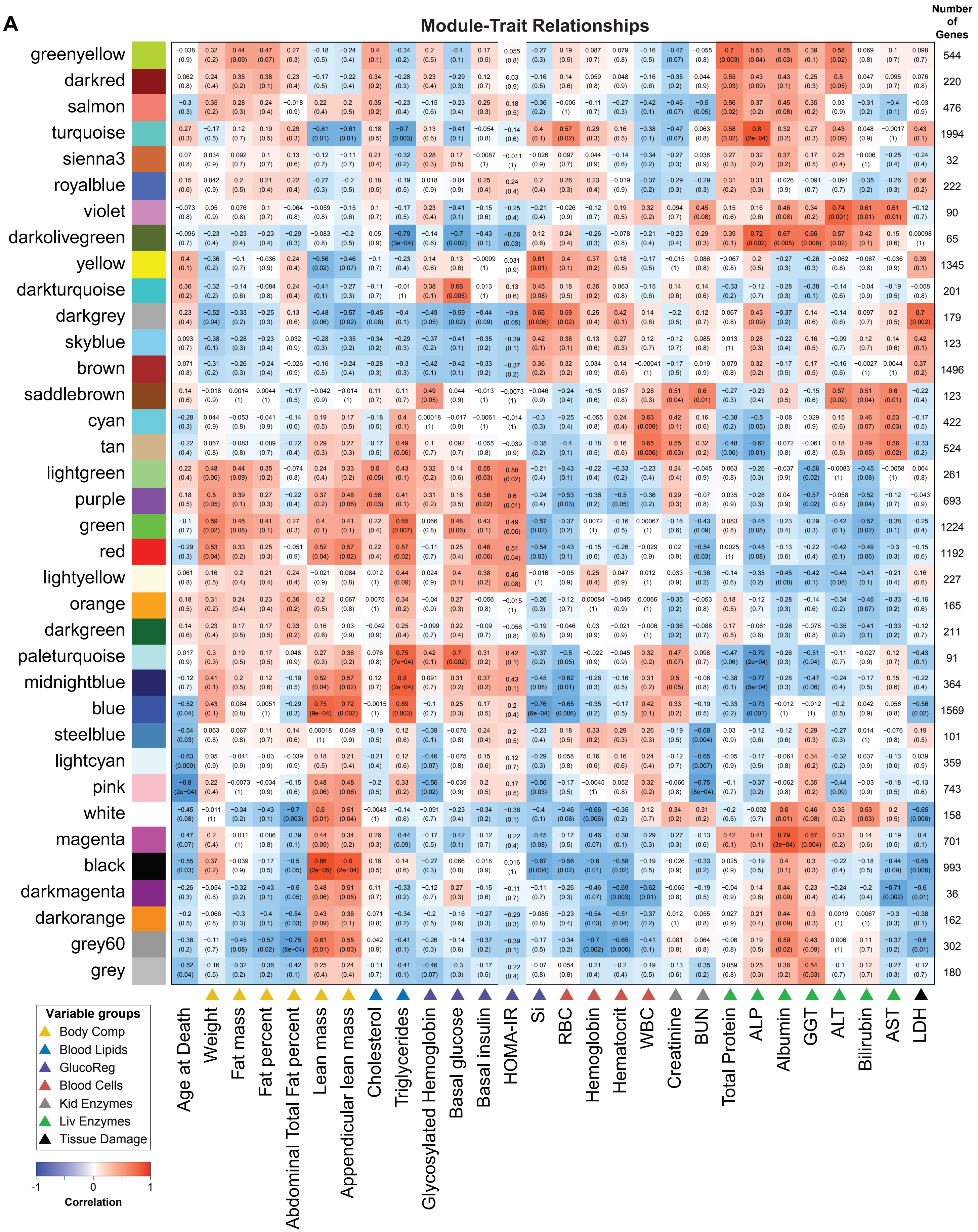

Supplementary Figure S4 (Related to Figure 4)

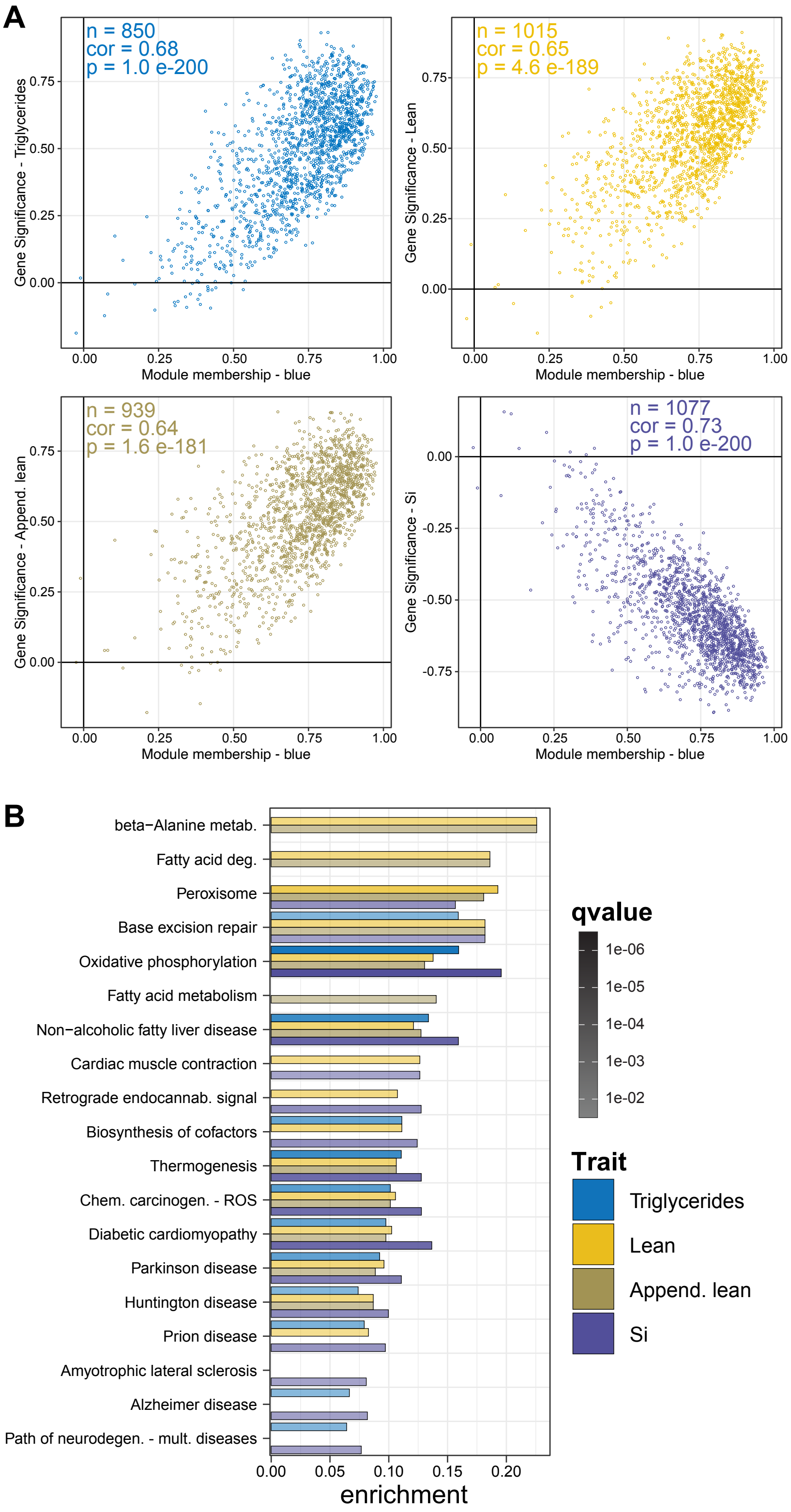

Supplementary Figure S5 (Related to Figure 4)
